# Biographical Sketches of Distinguished Living New York Surgeons

**Published:** 1866-05

**Authors:** 


*Biographical Sketches of Distinguished Living New York Surgeons.* By Samuel W. Francis, M. D., Fellow of the New York Academy of Medicine. New York: Published by John Bradburn. 1866.

The above is the title of a neat little volume of 220 pages, giving the principal facts connected with the individual history of the following named Surgeons:

Valentine Mott, William H. Van Buren, Alfred C. Post, Frank

H. Hamilton, James R. Wood, Lewis A. Sayre, Alexander B. Mott, John P. Batchelder, Alexander H. Stevens, Willard Parker, Gurdon Buck, John Swinburn, Julius Stephen Theband, Stephen Smith and Alexander E. Hosack.

